# Methods of assessment of patients for Nd:YAG laser capsulotomy that correlate with final visual improvement

**DOI:** 10.1186/1471-2415-4-13

**Published:** 2004-09-23

**Authors:** Tariq M Aslam, Niall Patton

**Affiliations:** 1 Manchester Eye Hospital, Manchester, UK; 2 Edinburgh Eye Pavilion, Edinburgh, UK

## Abstract

**Background:**

This paper attempts to clarify the usefulness of various simple pre-operative measures in estimating the potential for a visually successful capsulotomy.

**Methods:**

24 patients attending for capsulotomy had pre-operative measures of glare with BAT tester, visibility of posterior pole and grading of posterior capsular pearls and fibrosis seen at slit lamp. Visual function was measured before and after standardised capsulotomy. Correlations of the various preoperative measures with eventual visual function improvements were calculated.

**Results:**

Pearls at slit lamp and poor posterior pole visualisation were all correlated with improvements in visual acuity and contrast sensitivity after capsulotomy. Amount of fibrosis visible at slit lamp and glare assessment were not correlated with vision improvements after laser.

**Conclusion:**

Of the various measures that are taken prior to Nd : YAG capsulotomy, some correlate with eventual visual improvement but for others no clinical utility was found. Practitioners should note these findings as they are especially of use in more questionable or high-risk cases to help determine whether referral for PCO treatment by Nd: YAG capsulotomy is likely to benefit the patient.

## Background

Posterior capsular opacification (PCO) remains one of the most common post operative morbidities in modern day cataract surgery [1,2] and Nd:YAG posterior capsulotomy is one of the most commonly performed surgical procedures.

However, the Nd: YAG capsulotomy procedure has been associated with complications such as damage to intraocular lenses [3], post operative intraocular pressure increases [4], cystoid macular oedema [4], disruption of the anterior vitreous face [5] and increased incidence of retinal detachment [6].

Until recently Nd:YAG laser treatments have cost the U.S healthcare system up to $250 million annually [7]. Apart from exposing a patient to unnecessary risk, unqualified capsulotomies worsen this burden to the developed and developing world [8].

PCO is an extremely common development in patients after cataract extraction and in many mild cases it may not be immediately obvious whether it is visually significant. Patients may have reduced vision from other undetermined causes or have some measures of visual function that are not reduced at all.

Information is needed that will help the practitioners to decide on the visual significance of a patient's PCO using tools that are easily available.

The specific aim of this study was to assess the correlation between preoperative measures that are easily performed (fundus visibility, capsule opacity grading and initial glare testing) with eventual visual function improvements after Nd: YAG capsulotomy. This information would be of use to practitioners when deciding whether a patient had PCO that was clinically significant. In other words, we aimed to test the clinical utility of these preoperative tests for Nd:YAG laser capsulotomy.

## Methods

Local research ethical committee approval for the study was obtained and the study was performed in accordance with the treaty of Helsinki. 26 consecutive patients who had been referred for Nd: YAG capsulotomies for posterior capsular opacification were recruited and full informed consent was obtained. Patients excluded were those that had media opacities other than PCO or were not suitable for capsulotomy treatment. Patients in whom macular or disc pathologies were present were excluded from posterior pole assessment arm of the study as grading of visualisation would not be valid. Patients were roughly equally divided between silicone and acrylic IOLs.

Prior to Nd:YAG capsulotomy, the patients visual function was assessed with their normal physiological pupil state in terms of best corrected distance and near visual acuity using Bailey Lovie logMAR charts and contrast sensitivity with and without glare, using a Pelli-Robson chart under standard illumination levels at 1 meter. Glare was tested using a Mentor brightness acuity tester (BAT) instrument (set at medium illumination levels) and recorded as the level of contrast sensitivity chart read when exposed to the BAT.

Pupils were dilated with topical 1% tropicamide. After a minimum of 20 minutes, the size of the pupil was recorded using a millimetre ruler and the PCO was graded according to the slit-lamp appearance (grade 0 to 4). Use of such slit lamp grading has been established in scientific literature for PCO assessment [9-16] There are of course many other systems available for assessment of PCO [17] We chose a system based on slit lamp grading as it is the most commonly performed method of PCO assessment in practice. Many such slit lamp grading systems exist, but none have been proven to be gold standard in terms of clinical utility. After our own studies on the effect of PCO on visual function [18], we decided to use a protocol based on that described by Sellman and Lindstrom [19], recording pearls and fibrosis separately, which we feel is likely to be as clinically valid as any system that is commonly used in pre-assessment for Nd;YAG capsulotomy. All these tests were performed by one practitioner (T.A.), who was masked to fundus gradings, using the following scale, for pearls and fibrosis separately;

0 None visible at all

1 Visible but none reaching to IOL edge

2 At IOL edge

3 Well Inside IOL edge but visual axis clear

4 Across visual axis

Visualisation of the posterior pole was then assessed by examining the optic disc and macula using a Volk 90D lens. Visualisation of the optic disc was subjectively graded according to the following scale (adapted from the Madurai Intraocular lens study IV [20]):

0 Clear view of optic disc margin, blood vessels at the optic disc and nerve fibre layer (NFL examined using the red-free filter)

1 Clear view of optic disc margin, but disc blood vessels and/or nerve fibre layer are not clearly seen

2 Optic disc margin, as well as disc blood vessels and nerve fibre layer are not clearly seen

Visualisation of the macula was subjectively graded according to the following scale:

0 Clear view of foveal reflex, peri-foveal blood vessels and nerve fibre layer

1 Diminished foveal reflex, but clear view of peri-foveal blood vessels and nerve fibre layer

2 Blurred foveal reflex, peri-foveal blood vessels and/or nerve fibre layer

The totals for the visualisations of the optic disc and the macula were combined to produce a total posterior pole visualisation score (**PolVS**), ranging from 0 to 4 in order of decreasing visualisation.

All examinations of the posterior pole were carried out by the same examiner (NP). In order to be masked as to whether the patient was pre- or post- Nd:YAG capsulotomy, NP examined the fundus with the lenses already placed in front of the eye, thus obscuring any anterior segment view.

The patients then had Nd:YAG capsulotomy by the same surgeon (TA). This involved initial setting of 1 mJ and subsequent rises of 0.5 mJ as necessary to pierce the posterior capsule. The laser treatment was initiated off axis in a horizontal line across centre, followed by a line in the vertical axis to form a cross. Any obvious lines of capsule tear were treated with laser if deemed beneficial and overall energy used was kept to a minimum. Treatments in this manner produced small capsulotomies of size 2–3 mm diameter. Size of capsulotomy was dictated by ease of making openings, and concerns over energy used. In general the aim was to create an opening using minimum energy, which might be small, but which could be enlarged at follow up visits if deemed necessary.

Four weeks post Nd: YAG capsulotomy, the patients were reassessed again in terms of visual function as described earlier, by the same practitioner (TA). The pupils were dilated using 1% tropicamide and after a minimum 20-minute interval, the size of the pupil was recorded. The PolVS was again graded according to the same scales as before, by the same examiner (NP), again masked as to the state of the posterior capsule. Four weeks post-capsulotomy was chosen for re-examination, on the basis of evidence suggesting that capsulotomies enlarged progressively up to one month after Nd: YAG laser and then stabilised thereafter [21]

On inspection, the data was found to be of skewed distribution. All correlation calculations were therefore performed using the Spearman's rank correlation coefficients. Means were compared using Wilcoxon signed ranks test for non-normally distributed data. Significance was at the p < 0.05 level. Because the visualisation scale was composed of a combination of sub-scales (2 items for PolVS), internal validity was determined by the Cronbach test of reliability19,20. This is a commonly used test statistic to determine the degree with which constituent items within a scale correlate with each other. An alpha coefficient of = 0.7 is considered necessary for a composite of measurements to be considered a scale. Statistical analysis was performed using SPSS for Windows (version 8.0) for all calculations.

## Results

A total of 26 eyes of 26 patients were recruited into the study. Mean age was 75.2 years (range 52 to 90). There were 14 females and 12 males. 2 patients only had one examination pre-Nd:YAG capsulotomy, and declined any further examinations. Therefore, 24 patients were seen pre- and post- Nd:YAG capsulotomy. The PolVS scale was found to be internally reliable and consistent (α coefficient = 0.7824). Mean "pearl" grading score was 3.3 (± 1.3) and mean "fibrosis" grading score was 2.3 (± 1.4).

5 patients were omitted from PolVS score as macular or disc pathology prevented objective grading. Mean improvement of PolVS (n = 19) was 1.95 (S.D. ± 1.31) (95% C.I. 1.32 to 2.58) (p < 0.0001, Wilcoxon signed ranks test).

**Figure 1:**
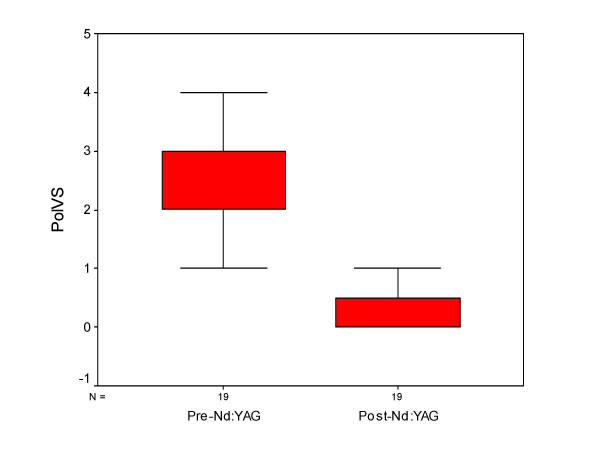
Boxplots of PolVS pre- and post-Nd:YAG capsulotomy.

Improvements in the PolVS score after Nd: YAG capsulotomies are shown in fig. 1.

Mean improvement of distance logMAR visual acuity was 0.32 (± 0.29) (95% CI 0.20 to 0.44) and near logMAR visual acuity was 0.32 (± 0.29) (95% CI 0.20 to 0.44). Mean improvement of log contrast sensitivity was -0.41 (± 0.39) (95% CI 0.25 to 0.57) and glare testing vision was -0.35 (S.D. ± 0.41) (95% CI 0.18 to 0.52). (Improved contrast sensitivity and glare testing visual function are associated with lower grading scores.) For all the above analyses, p < 0.0001, except for mean improvement in glare testing (p = 0.001) (Wilcoxon signed ranks test).

The main aim of the study was to determine which pre-operative assessments on patients attending for Nd: YAG capsulotomy would correlate with eventual visual function outcome of the patient, and thus be useful clinical measures. The results of the various correlation coefficient analyses are shown in table 1. The table demonstrates that a patient's improvement in visual function after laser capsulotomy is correlated to measurements made before treatment of pearls at slit lamp and posterior pole visibility score.

Measurements of fibrosis at slit lamp and of glare do not correlate with eventual improvements in vision after capsulotomy.

## Discussion

If it is decided to offer a patient Nd; YAG capsulotomy treatment they need to be told of the likely benefits of the treatment as well as risks. Information on potential visual function improvements for each specific patient would be especially welcome in decisions in uncertain cases and higher risk patients such as high myopes.

Practitioners should have some evidence for the potential benefit to visual function before suggesting treatment. Retroilluminated photograph analysis by computer have been correlated with improvements in vision [22] but these tools are not available to most ophthalmologists in the examination room. Practitioners are able to subjectively estimate the amount of PCO on slit lamp examination of posterior capsule but often with more subtle PCO it is difficult to assess clinical relevance. It is thought by some [23] that contrast sensitivity with glare testing are likely to be particularly sensitive to visual loss from PCO, but of course these visual parameters are also open to influence from many other ocular states. Visibility of discs has been used as an assessment of the amount of PCO [20] but without convincing evidence of validity.

Although all of the above tests have been used to try to assess how much benefit a particular patient may gain from capsulotomy, there has previously been little empirical evidence supporting their use. This study provides some evidence for the use and avoidance of various preoperative tests in assessing potential benefit from Nd; YAG laser capsulotomy.

### Preoperative glare measures

There has been some controversy as to the relative usefulness of the different measures of visual function for assessing PCO severity [23-27], with some suggestion that glare assessment might be particularly useful for such anterior segment disorders [23,27] This study suggests that initial glare measures with the BAT did not correlate with eventual improvements in any visual function. Our results do, however, show a definite improvement in glare following Nd:YAG capsulotomy, all of whom underwent a small capsulotomy (2–3 mm). It appears that glare assessment with BAT is of no clinical utility when assessing the potential improvement a patient may gain from Nd:YAG capsulotomy. This may reflect the difficulties of BAT glare measurement which has been found to be inconsistent in some studies [28]. Indeed, glare assessment by any means is not simple to perform reliably and with clinical utility.

### Slit lamp assessment of PCO by practitioner

There were two types of PCO assessed, pearls and fibrosis, both graded 0–4 with identical criteria for severity. The study shows very clearly that the fibrosis score was not significantly correlated to any improvements in visual function. The pearl score however was significant in correlating with the eventual improvement of all visual functions. This agrees with previous work using computerised analysis of retro-illumination photographs, which showed that only very central fibrosis affected vision whereas pearls could be detrimental even in para-axial locations [18] Other studies have also shown that pearls have a greater effect on vision than fibrosis [29].

The assessment of fibrosis at slit lamp is commonly performed before Nd:YAG capsulotomy, but according to this study does not correlate with eventual visual outcomes. This finding was perhaps the most poignant due to the common clinical use of amount of fibrosis at slit lamp when deciding whether a patient would benefit from capsulotomy. The finding deserves further investigation, but it may be that antero-posterior thickness of the fibrosis as well as density of opacification are important contributory variables to any visual loss. This thickness of PCO would not be easily assessed accurately and routinely at slit lamp, and was not incorporated into the grading scale used for this experiment.

In contrast, clinical utility of pearl assessment is demonstrated, by its correlation with eventual improvement of visual function.

### Visualisation of posterior pole

Visualisation of posterior pole according to the scale above correlated with improvements in visual acuity and contrast sensitivity. Using the specific criteria as described may have increased the objectivity of the test. This confirms expected agreement between the difficulty of visualization of the posterior pole and the patient's own vision of the outside world.

An assessment of the posterior pole along the suggested guidelines is shown to have significant clinical utility.

It may be that some of the correlations found were due to some criteria measured being closely related, for example, pearls at sit lamp and visualisation of fundus. The numbers in this analysis were unfortunately insufficient for stepwise multiple linear regression analysis, which could be used in future studies to determine the relative importance of the different measures. However the principle aims of the current study were realised with the presented experimental protocol and statistical analysis.

## Conclusions

This study confirms the clinical value of pre-YAG measurements of pearls graded at slit lamp and posterior pole visualisation.

However, other measures were not significantly correlated with improvements in visual function, namely pre-YAG testing of glare with BAT and pre-YAG grading of fibrosis seen at slit lamp.

If a practitioner is uncertain as to the potential visual benefit of an Nd:YAG capsulotomy, an assessment of posterior pole visualisation or pearls at slit lamp should be useful. There is little clinical utility shown from this study in attempting glare assessment or assessment of fibrosis at slit lamp and these assessments may lead to erroneous expectations from treatment.

This information is of practical value to practitioners faced with the common problem of assessing suitability of patients for Nd: YAG capsulotomy. It should especially be of use in more uncertain or high-risk cases to determine whether referral to PCO treatment by Nd: YAG capsulotomy is clinically appropriate.

## Competing interests

The author(s) declare that they have no competing interests.

## Authors' contributions

Both authors were involved in planning, design, execution and writing of this paper. Both read and approved the final manuscript.

## Pre-publication history

The pre-publication history for this paper can be accessed here:



## Figures and Tables

**Table 1:** Correlation cross-tabulation table between improvements in visual function and pre-treatment measures of glare, slit-lamp pearls, slit-lamp fibrosis and posterior pole visibility score (Polvs).

	Improvement in Distance Visual Acuity	Improvement in Contrast Sensitivity
Pre-Nd:YAG	R = -0.30	R = -0.35
Glare Testing	p = 0.159*	p = 0.093*
Slit-lamp	**R = 0.67**	**R = 0.64**
Pearls	**p < 0.0001**	**p = 0.001**
Slit-lamp	R = -0.01	R = 0.06
Fibrosis	p = 0.950*	p = 0.775*
Pre-Nd:YAG	**R = 0.56**	**R = 0.50**
PolVS	**p = 0.012**	**p = 0.029**

R = Spearman's Rank Correlation Coefficient

* = Not statistically significant
